# Use of Almond Milk, Almond Skin and Plant Based Probiotics on Newly Developed Kefir: Physical, Chemical, Microbiological, and Functional Properties

**DOI:** 10.1002/fsn3.70719

**Published:** 2025-07-27

**Authors:** Ayşe Şahin, İlkay Buran, Ceren Akal

**Affiliations:** ^1^ Department of Food Toxicology Karabük University Institute of Graduate Programs Karabük Turkiye; ^2^ Faculty of Agriculture, Department of Dairy Technology Ankara University Ankara Turkiye

**Keywords:** almond milk, almond skin, plant‐based kefir

## Abstract

Plant‐derived food products are among the important nutrients that form the basis and sustainability of human nutrition. In this study, almond milk was obtained from raw almonds, and a plant‐based kefir was produced with a plant‐based kefir starter culture. The physicochemical properties, color analysis, mineral content, organic acid, total phenolic compound content, antioxidant activity, microbiological properties, rheological properties, and sensory evaluation of the almond kefir samples were determined on the 1st, 7th, and 14th days of storage. According to the findings, while an increasing trend was observed in antioxidant activity, total phenolic compound content, and microbiological properties with the increase in the skin ratio, it was determined that acceptability decreased with the increase in the skin ratio in sensory evaluation. Increasing the skin ratio in almond kefir influenced various physical, chemical, and microbiological properties, and these effects also varied with storage time. A high added‐value product in terms of antioxidant and phenolic compounds was obtained in samples enriched with almond, a functional dairy product. Considering that almond milk is rich in fiber content and almond skin can also have a prebiotic effect, almond kefir is thought to be a synbiotic drink that regulates intestinal microbiota. This innovative product is thought to be a healthy alternative for vegans, those with lactose intolerance, and cardiovascular patients.

## Graphical abstract

In this study, in order to obtain almond kefir, a functional dairy product, almond inner skin was separated, dried and powdered in the first stage. In the second stage, milk was obtained from raw almonds and kefir was obtained by adding 1%, 3% and 5% powdered almond skin. The physicochemical properties, color analysis, mineral content, organic acid, total phenolic compound content and antioxidant activity, microbiological and sensory properties of the samples were examined during the 14‐day storage period.

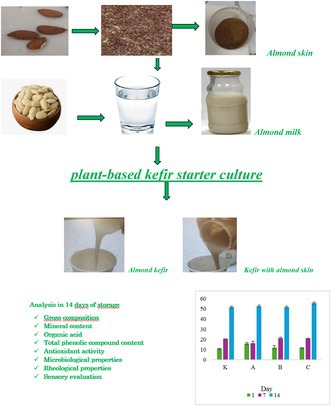

## 1 Introduction

Functional foods are increasingly produced to meet growing consumer demand for bioactive compounds. This trend has led to the development of innovative nondairy products based on plant sources such as coconut, oats, and almonds. The food processing industry has shifted towards producing nutrient‐enriched foodstuffs from plant sources in response to consumer preferences for plant‐based diets. Plant‐based diets, especially vegan diets, are gaining popularity worldwide due to their potential to promote optimal health and prevent chronic diseases when balanced and varied. Plant‐based milks, primarily produced from oilseeds and cereals, are recognized as foods with functional properties and have seen increased global consumption (Bernat et al. 2014). This rise is attributed to the growing prevalence of cow's milk protein allergies, lactose intolerance, hypercholesterolemia, and interest in vegan diets (Sethi et al. 2016). While soya and oat‐based milks have been popular, recent years have seen increased production and consumption of oilseed‐based milks such as peanut, almond, hazelnut, walnut, and cashew.

Plant‐based milks are considered functional foods and nutraceuticals due to their bioactive components beneficial for health. These milk alternatives can be used to create fermented products like yogurt and cheese by adding probiotic microorganisms, providing suitable options for vegan and vegetarian diets. However, it is crucial that the added probiotics are derived from plant sources and do not contain animal‐derived components.

Almond ( *Prunus amygdalus* ), a tree species belonging to the Prunoideae subfamily within the Rosaceae family, is widely used in plant‐based functional food production. Studies have shown that phenolic compounds in fermented soya and almond milk may reduce the occurrence of oxidative stress‐related diseases such as cancer, coronary heart disease, and atherosclerosis (Wansutha et al. 2018). Almonds have higher protein bioavailability compared to other plant‐based proteins, with a total protein content of 21.2% and high arginine content. Their low sugar content (about 3.9%) qualifies them as a “naturally low sugar” food according to European Union regulations (European Parliament and Council 2007). Almonds also contain approximately 12% dietary fiber (Mandalari et al. 2008). The low glycemic index of almonds is attributed to their low carbohydrate content, healthy fatty acids, high plant‐based protein content, dietary fiber, and magnesium. The positive effects of almonds on cholesterol and cardiovascular health are due to their high content of dietary fiber, potassium, calcium, magnesium, tocopherol, phytosterol, polyphenolic compounds, and appropriate unsaturated‐saturated fat ratio (Richardson et al. 2009). Phytosterols in almonds also inhibit intestinal cholesterol absorption. Studies have reported that consuming 100 g of almonds daily reduces total cholesterol and LDL cholesterol (Williams et al. 2019).

Almond hulls are industrially removed from almonds by scalding with hot water and constitute 4%–8% of the total weight of skin almonds (Milbury et al. 2006). The almond skin (seed coat) surrounding the almond is removed and discarded after blanching. The total fiber, fat, and protein values in the skin are 47.5%, 22.2%, and 12.8% in natural and 45.1%, 24.2%, and 10.3% in boiled almonds, respectively (Mandalari, Tomaino, et al. 2010). In addition, although the almond skin constitutes about 45%–8% of almonds, it has been reported to contain 60%–80% of total phenolic compounds in recent studies. Phytochemicals such as phytosterol, phenolic acid, and polyphenolics are more concentrated in the skin of almonds (Chen et al. 2006). As the fiber in the almond skin consists of plant cell wall polysaccharides that are not degraded by endogenous enzymes in the upper gastrointestinal tract, it is very important for the growth of beneficial bacteria in the large intestine (Mandalari, Faulks, et al. 2010). At the same time, these polysaccharides in the cell wall of almond skin have an important role in colonic function by fermentation in the large intestine, maintaining intestinal habit, transit times, metabolism, balance of commensal flora, and large intestinal epithelial health (Mandalari, Faulks, et al. 2010). Flavonoids in almond skin are beneficial for health. Flavonols and flavan‐3‐ols are reported to have beneficial effects on the gastrointestinal system as well as antiviral, anti‐inflammatory, antiallergic, antimutagenic, anticarcinogenic, and anticholesterolemic activities (Brahmachari and Gorai 2006). In a study investigating the prebiotic effects of almond and almond skin intake in healthy people, it was emphasized that *Bifidobacterium* spp. and *Lactobacillus* spp. populations increased significantly after the consumption of almond skin or almonds and that bifidobacteria and lactobacilli increased faster in individuals consuming almond skin compared to individuals consuming almonds. It was also reported that the skin has prebiotic potential similar to prebiotic fructooligosaccharides (Liu et al. 2013).

Kefir is an acidic beverage with low alcohol content, formed as a result of fermentation of kefir grains with water or milk (Garofalo et al. 2020). Aside from animal sources such as cow, sheep, goat, and camel milk used in kefir production, it can also be produced from plant sources such as soya milk, rice milk, coconut milk, and almond milk. In a study comparing almond kefir with kefir obtained from cow, cashew, hazelnut, pistachio, and walnut milk, it was reported that almond milk kefir was the best example in terms of microbiological and functional properties (antioxidant capacity, phenolic content) (Çomak Gocer and Koptagel 2023). However, no research has been conducted on the use of plant‐based kefir culture in kefir production. Therefore, with the idea that the innovative functional beverage would be a new product alternative for vegan individuals, in this study, a plant‐based kefir beverage was produced by adding almond skin and plant‐based kefir culture to milk obtained from raw almonds. For this purpose, some physical, functional, and bioactive properties of almond kefir samples produced by enriching them with powdered almond skin at different rates (1%, 3%, and 5%) were evaluated during 14 days of storage.

## 2 Material and Methods

### 2.1 Materials

Raw almonds were purchased from a local supplier (Tarihi Elazığ Leblebicisi, Elazığ, Turkiye). A plant‐based kefir starter culture was obtained from Danem Süt Ürünleri (Isparta, Turkiye). The starter culture contained *Lactobacillus* (8.96 log cfu/mL), *Lactococcus* (9.40 log cfu/mL), total yeast (3.0 log cfu/mL), *Lactobacillus acidophilus* (4.84 log cfu/mL), and *Bifidobacterium* (4.46 log cfu/mL).

### 2.2 Milk Production From Almond

Almond milk production followed a modified procedure described by Ustaoğlu‐Gençgönül (2022). Raw almonds were soaked overnight in a 1:3 ratio with drinking water, drained, and peeled. The skins were dried at 37°C for 3 days and ground twice. Almond milk was prepared by blending the peeled almonds with potable water in a 1:2 ratio for 1–2 min using a Kenwood Thermoresist Glass Blender AT338 (Havant, UK). The resulting mixture was filtered twice through two layers of cheesecloth. Almond milk and kefir samples were produced at Ankara University Department of Dairy Technology.

### 2.3 Kefir Production From Almond's Milk

**FIGURE 1:**
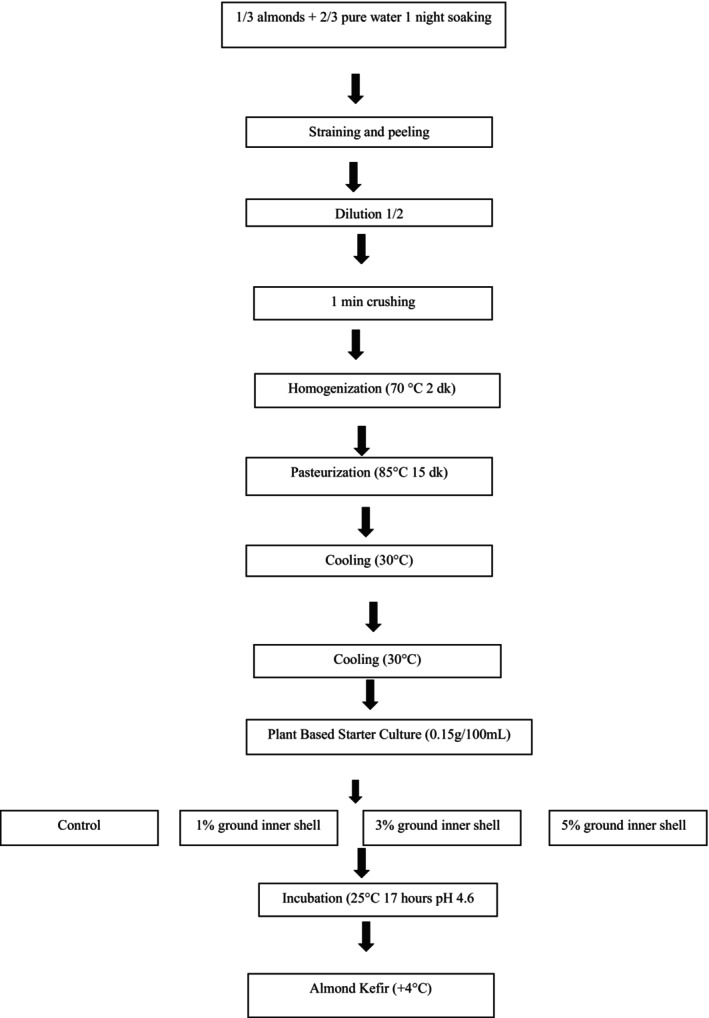
Plant‐based kefir production scheme from almond milk.

For kefir production, almond milk was preheated to 40°C and supplemented with 4% maltodextrin, 1% sugar, 0.1% salt, and 1% stabilizer (gum arabic). The milk was blended and homogenized using an ultraturrax (T‐25; IKA, Staufen, Germany) at 8000 rpm. After homogenization at 70°C for 2 min, the mixture was pasteurized at 85°C for 15 min in a water bath (Heto Shaking SBD50‐1 Bio Comfort Heated Water Bath Germany). Upon cooling to 30°C, plant‐based kefir starter culture (0.15 g/100 mL) was added. Non‐control samples were fortified with powdered almond skins at 1%, 3%, and 5% concentrations. Samples were incubated at 25°C for approximately 17 h until reaching a pH of 4.6. The kefir samples were then packaged in 200 mL steril bottles to prevent contamination and kept in the refrigerator at 4°C. Analysis was performed at the 1st, 7th, and 14th day of storage. The almond milk kefir production flow chart is shown in Figure 1 (Ustaoğlu‐Gençgönül 2022). Sample codes are as shown below.

K: (0.15% plant‐based kefir starter culture +1% Sugar +0.1% salt +4% maltodextrin +1% gumarabic). A: (0.15% plant‐based kefir starter culture +1% Sugar +0.1% salt +4% maltodextrin+1% gum arabic + 1% almond skin). B: (0.15% plant‐based kefir starter culture +1% Sugar + 0.1% salt + 4% maltodextrin+1% gumarabic +3% almond skin). C: ((0%) 15 plant‐based kefir starters +1% sugar +0.1% salt +4% maltodextrin +1% gum arabic +5% almond skin).

### 2.4 Physicochemical Analyses

Total solids and ash contents of almond milk and kefir samples were analyzed by the gravimetric method at 100°C ± 2°C on the 1st day of storage using the Association of Official Analytical Chemists–AOAC (2000) standard method. Titratable acidity of samples, which was expressed as lactic acid %, was measured by titration with 0.1 N NaOH (Merck, Darmstadt, Germany) following the Association of Official Analytical Chemists–AOAC (2000) standard method. Fat content was measured by the Gerber‐Van Gulik method (Anonymous 1990) total nitrogen (TN) was carried out by applying the Kjeldahl method as described by IDF (2001). Energy content was calculated by multiplying the mean values of protein, fat, and carbohydrate by 4, 9, and 4, respectively, as per Gibson (1990). pH values of each sample were measured using a pH meter (Seven Compact, Mettler Toledo, USA). Mineral content in almond milk was determined using ICP‐OES (Spectro Analytical Instruments, Kleve, Germany) following the method described by Ustaoğlu‐Gençgönül (2022). Samples were incinerated using the CEM MARS6 model Microwave Incineration System for Extraction. Then, 10 mL HNO3 (67% v/v) was added to the sample. The program was set for combustion. At the end of the process, the solutions were allowed to cool to room temperature. The samples were then topped up with ultrapure water. The prepared samples and calibration solutions were analyzed in the SpectroBlue brand ICP‐OES device to determine the mineral matter concentrations.

### 2.5 Organic Acid Analysis

Organic acid analyses were conducted following the method described by Bulat and Topcu (2020). Samples (3 g each) were mixed with 30 mL of 0.013 N H_2_SO_4_ and homogenized using an ultraturrax. The mixture was centrifuged at 10,000 × g for 20 min at 4°C. The supernatant was filtered through a 0.45 μm syringe filter before HPLC injection. The HPLC system (1100 HPLC System; Agilent Technology CA, USA 1100 series) equipped with a PDA detector and a C18 column 4.6 cm × 250 mm × 5 μm particle size at 65°C oven temperature; was used for analysis. A 20 μL sample was injected, and the mobile phase consisted of 0.013 N H_2_SO_4_ at a flow rate of 0.5 mL/min. Organic acid peaks were detected at 210 nm. Citric acid, lactic acid, and acetic acid were used as standard solutions to determine the organic acid content of the samples (Sigma‐Aldrich). The concentrations of the standard solutions were monitored in the range of 100 ppm–1600 ppm, and the concentrations (mg/L) of the samples were determined using the graphs obtained.

### 2.6 Microbiological Analyses

Microbiological analyses were conducted using various selective media and incubation conditions. Total mesophilic aerobic bacteria (TMAB) were enumerated using Plate Count Agar (PCA) (Merck, Darmstadt, Germany) with aerobic incubation at 30°C for 48 h. *Lactococci* counts were performed on M17 agar (Merck, Darmstadt, Germany) at 30°C for 72 h. *Lactobacilli* were enumerated on De Man Rogosa Sharp (MRS) agar (pH 6.5 ± 0.2; Merck, Darmstadt, Germany) under anaerobic conditions at 30°C for 72 h.

Yeasts were counted on potato dextrose agar (PDA) containing 0.10 mL/100 mL tartaric acid after incubation at 25°C for 5 days. *Lactobacillus acidophilus* La‐5 was enumerated on MRS‐sorbitol agar (using 10 g/L sorbitol) after anaerobic incubation at 37°C for 72 h. *Bifidobacterium animalis* Bb‐12 populations were counted on MRS agar supplemented with NNPL (neomycin sulphate 100 mg/L, nalidixic acid 15 mg/L, paromomycin sulphate 200 mg/L, lithium chloride 3 mg/L, and l‐cysteine chloride 0.5 mg/L; Sigma‐Aldrich, St. Louis, MO). These plates were incubated anaerobically at 37°C for 72 h.

### 2.7 Determination of Antioxidant Activity

Antioxidant capacity was determined according to the 2.2‐diphenyl‐1‐picrylhydrazyl (DPPH) method. Each sample (5 mL) was taken, and 5 mL of ethanol (80%) was added, mixed with a vortex for 1 min, and centrifuged at 4000 g for 20 min. The supernatant (0.1 mL) was filtered, and 3.9 mL of DPPH (0.1 mM) was added. After 40 min in the dark, a reading was performed at 517 nm wavelength using a spectrophotometer (Lambda 25 UV/Vis, PerkinElmer, Singapore) (Blois 1958).

(2) $$\text{Antioxidant Activity}\%=\frac{A_{\text{blank}}-A_{\text{sample}}}{A_{\text{blank}}}\times 100$$

### 2.8 Determination of Phenolic Compounds

The modified Folin‐Ciacalteu method was used for the determination of total phenolic content of the samples (Şahin et al. 2013). Each sample (5 mL) was taken, and 5 mL of 80% methanol was added and mixed with vortex for 1 min. After centrifugation at 3500 g for 45 min, it was filtered with filter paper. These samples (0.4 mL), 2 mL of folin, and 1.6 mL of 75% sodium carbonate (Na_2_CO_3_) were added to the tubes and kept in the dark for 1 h. A reading at 765 nm was taken in a spectrophotometer (Lambda 25 UV/Vis, PerkinElmer, Singapore). The results were calculated according to the formula obtained from the calibration graph over the gallic acid equivalent (μg GAE/g).

### 2.9 Determination of Color Parameters

The color of kefir samples L*, *a*, * and b* was determined using a Chroma Meter, CR‐400, (Minolta, Osaka, Japan) colorimeter. The following formula was used for the total color difference.

(3) $$\Delta E=\surd \left({\left(\Delta L^{*}\right)}^{2}+{\left(\Delta a^{*}\right)}^{2}+{\left(\Delta b^{*}\right)}^{2}\right)$$

### 2.10 Dynamic Rheological Tests

The dynamic rheological properties of kefir samples were measured with Malvern brand Kinexus Pro+ model rheometer. The samples were stirred at a constant speed for 30 s at 5°C. Rheological measurements of the almond kefir samples were carried out at 5°C and at a shear rate range of 0.1–100 s^−1^ with a 4° angled conical probe. Measurements were made at 31 different shear rates. The data obtained were interpreted with the Herschel‐Bulkley model (Akal 2017).

### 2.11 Sensory Analyses

The hedonic type scale applied by Meilgaard et al. (2016) was used for the sensory evaluation of kefir samples. Consisting of 7 trained panelists (30–45 age between and 5 female/2 male), the sensory analysis was carried out by scoring from 1 to 9 (1 = bad, 9 = very good) in terms of appearance, body, and taste. Approximately 100 mL of the kefir samples were served in the transparent plastic cups to the panelists.

### 2.12 Statistical Analyses

A randomized complete block design was applied for four treatments, three storage periods, and two trials, to analyze the variables relating to the characteristics of fermented almond's milk. Analysis of variance (ANOVA) was performed in the statistical evaluation of the results using the Minitab package (Minitab 20,4; Minitab Inc., State College, PA) in a randomized complete block design. Tukey's multiple comparison test (*p* < 0.05 level) was used to determine the different groups.

## 3 Results and Discussion

### 3.1 Physicochemical Properties

The physicochemical properties of raw almond milk and kefir samples on the 1st day of storage are presented in Table 1. Samples A, B, and C, which contained added almond skin, exhibited higher total solid content than the control sample (K). Analysis of fat content in almond kefir samples revealed that sample K had the highest fat content, followed by samples A, B, and C, respectively. This difference, likely due to the composition of the almond skin, was not statistically significant. In our study, the protein value of almond kefir samples was determined in the range of 5.59%–6.43%. Similarly, Hew et al. (2023) reported that almond kefir had protein values of 5.69%–5.87%. As the protein content of plant‐based kefir varies depending on the protein content of the raw milk and the fermentation process, close results can be obtained in similar products.

**TABLE 1:** Physicochemical properties of almond milk and kefir samples (mean ± SE; *n* = 2).

Composition of milk and kefir samples								
Type of product	Protein (%)	Fat (%)	Total solids (%)	Ash (%)	Na (mg/L)	Mg (mg/L)	Ca (mg/L)	K (mg/L)
Milk	4.83 ± 0.02	8.30 ± 0.00	14.00 ± 0.19	0.54 ± 0.01	—	—	—	—
Kefir								
K	6.03 ± 0.74	8.35 ± 0.35	20.16 ± 0.33^C^	0.32 ± 0.01^B^	470.14 ± 2.82	518.11 ± 1.14^B^	58.67 ± 0.47^C^	1428.17 ± 0.85^A^
A	5.49 ± 0.26	7.75 ± 0.75	21.37 ± 0.28^bc^	0.34 ± 0.01^B^	390.17 ± 0.66	444.13 ± 1.41^D^	62.50 ± 15.83^C^	1260.53 ± 51.68^B^
B	5.98 ± 0.30	7.15 ± 0.05	22.92 ± 0.27^AB^	0.65 ± 0.01^A^	427.75 ± 0.00	544.17 ± 1.12^A^	91.79 ± 0.65^B^	1403.29 ± 1.23^A^
C	6.03 ± 0.34	6.85 ± 0.15	24.27 ± 0.29^A^	0.69 ± 0.00^A^	332.33 ± 0.24	474.53 ± 0.56^C^	107.00 ± 0.35^A^	1181.08 ± 0.94^C^

*Note:* K (0.15% plant‐based kefir starter culture +1% Sugar +0.1% salt +4% maltodextrin +1% gumarabic). A (0.15% plant‐based kefir starter culture +1% Sugar +0.1% salt +4% maltodextrin + 1% gum arabic + 1% almond skin). B (0.15% plant‐based kefir starter culture +1% Sugar + 0.1% salt +4% maltodextrin +1% gumarabic +3% almond skin). C ((0%) 15 plant‐based kefir starters +1% Sugar +0.1% salt +4% maltodextrin +1% gumarabic +5% almond skin). Different capital letters in the same column indicate that the difference between the samples is significant (*p <* 0.05). The absence of letters indicates that there is no statistical difference between samples (*p* > 0.05).

A significant increase in ash content was observed in samples A, B, and C as the amount of skin increased (*p* < 0.05) (Malayil et al. 2022). All kefir samples were found to be rich in calcium (Ca), potassium (K), magnesium (Mg), and sodium (Na). The difference in Ca, Mg, and K contents between kefir samples was statistically significant (*p* < 0.05). Previous studies on almond milk (Ustaoğlu‐Gençgönül 2022) reported different mineral content compared to this study. Drogoudi et al. (2013) noted that mineral content in 72 almond varieties from three countries varied significantly depending on the almond type. Mineral intake is crucial for human health, and the Food and Drug Administration recommends daily doses of 3000 mg potassium and 1300 mg calcium for adults. Thus, food sources rich in these minerals are nutritionally important. Notably, sample C, containing the highest amount of almond skin, showed higher calcium content than the control sample, suggesting that almond skin is rich in calcium.

### 3.2 Acidity Values and Organic Acid Profile of Kefir Samples

The titratable acidity (TA), pH values, and organic acid profiles of kefir samples are presented in Table 2. It was determined that the control sample (K) had the lowest titratable acidity value during 14 days of storage. The acidity value in terms of lactic acid increased with the increase in the amount of almond skin. The findings showed that almond milk creates a suitable environment for the growth of plant‐based kefir culture. Having a higher TA value at Day 14 than at Day 7 in the samples may have a positive effect on the overall sensory properties of the samples, as this is a critical characteristic in kefir (Tamime and Robinson 1999). Due to the low carbohydrate content of almond drink (Bernat et al. 2014) the addition of carbohydrates such as almond skin is thought to have a positive effect on microorganism activity and increase acidity.

**TABLE 2:** Acidity and organic acid concentrations of almond kefir samples (mean ± SE; *n* = 2).

Samples	Storage (day)	Titratable acidity (LA)	pH value	Asetik asit (mg/L)	Laktik asit (mg/L)	Sitrik asit (mg/L)
K	1	0.81 ± 0.01^E^	4.36 ± 0.01^CD^	3641.83 ± 145.62^A^	19871.93 ± 451.10^AB^	2031.54 ± 367.46^EF^
	7	0.80 ± 0.00^E^	4.39 ± 0.02^bc^	3105.47 ± 253.00^B^	21341.33 ± 449.34^AB^	4104.65 ± 389.94^bc^
	14	0.85 ± 0.00^DE^	4.22 ± 0.02^E^	2802.60 ± 52.90^B^	21378.53 ± 1219.81^AB^	1391.27 ± 116.14^F^
A	1	0.84 ± 0.01^E^	4.48 ± 0.01^A^	3188.17 ± 109.97^B^	20317.67 ± 34.19^AB^	2389.61 ± 89.03^DEF^
	7	0.82 ± 0.03^E^	4.45 ± 0.02^AB^	4277.91 ± 424.77^B^	25070.75 ± 1423.05^A^	6101.85 ± 401.13^A^
	14	0.89 ± 0.01^BCDE^	4.22 ± 0.00^E^	3450.39 ± 23.80^B^	21827.87 ± 238.19^AB^	2879.76 ± 213.23^CDE^
B	1	0.98 ± 0.01^AB^	4.50 ± 0.00^A^	3875.13 ± 0.11^B^	21654.83 ± 460.66^AB^	3721.38 ± 252.74^BCD^
	7	0.84 ± 0.02^E^	4.48 ± 0.01^A^	3725.77 ± 204.98^B^	19677.73 ± 1683.24^AB^	3636.59 ± 119.11^BCD^
	14	0.94 ± 0.03^ABCD^	4.25 ± 0.00^E^	3756.26 ± 242.81^B^	21172.22 ± 238.19^AB^	3726.92 ± 300.79^BCD^
C	1	0.99 ± 0.02^A^	4.53 ± 0.03^A^	3717.22 ± 234.72^B^	22400.11 ± 1250.30^AB^	4376.56 ± 111.35^B^
	7	0.87 ± 0.03^CDE^	4.49 ± 0.02^A^	2489.10 ± 161.32^B^	16989.77 ± 1652.78^B^	3297.91 ± 232.51^BCDE^
	14	0.96 ± 0.02^ABC^	4.29 ± 0.02^DE^	3190.25 ± 195.68^B^	22199.35 ± 994.56^AB^	4402.20 ± 284.85^B^

*Note:* K (0.15% plant‐based kefir starter culture +1% Sugar +0.1% salt +4% maltodextrin +1% gumarabic). A (0.15% plant‐based kefir starter culture +1% Sugar +0.1% salt +4% maltodextrin +1% gum arabic + 1% almond skin). B (0.15% plant‐based kefir starter culture +1% Sugar + 0.1% salt +4% maltodextrin+1% gumarabic +3% almond skin). C ((0%) 15 plant‐based kefir starters +1% Sugar +0.1% salt +4% maltodextrin +1% gumarabic +5% almond skin). Interactions were detected between samples and storage days, and the differences between the values were shown with different capital letters (*p* < 0.05). The absence of lettering indicates that the difference between the samples is insignificant (*p* > 0.05).

The pH values of kefir samples were in the range of 4.22–4.53. The pH values of all samples decreased during 14 days of storage. The pH value increased as the ratio of almond skin added to the samples increased. The reason for this may be the high initial pH value of the samples due to the pH value of the added almond skin being close to neutral. However, the decreases in the samples during storage were similar to each other and had similar values on the last day of storage.

Organic acids are compounds that give foods their unique taste, increase their flavor, and help digestion. Organic acid contents of lactic acid, acetic acid, and citric acid were determined in kefirs produced as a result of fermentation of plant‐based almond milk with plant‐based kefir culture (Table 2). The highest amount of lactic acid was found in the almond kefir coded A on the 7th day of storage (16989.77–25070.75 mg/L), and the lowest amount of lactic acid was found on the 1st day of the control sample, and a significant difference was found between the samples and storage periods (*p* < 0.05). Some microorganisms in kefir microflora can produce an organic acid that other microorganisms can use as a substrate. This may affect the production of different metabolites, which may cause differences in the sensory properties of the final product (Bulat and Topcu 2021; Çomak Gocer and Koptagel 2023).

Acetic acid (2802.60–4277.91 ± 424.77 mg/L), which is a by‐product of citrate metabolism and is also produced in heterofermentative lactose metabolism through oxidation of lactic acid, differed between both samples and storage periods (*p* < 0.05). The highest amount of acetic acid was observed in A coded almond kefir on the 7th day of storage.

The citric acid level reached the highest value (6101.85 mg/L) on the 7th day of the A coded sample (containing 1% almond skin). Fermentation of carbohydrates used as substrate and hydrolysis of lipids have an effective role in the formation of different organic acids during the production and storage of fermented products (Çomak Gocer and Koptagel 2023). Some lactic acid bacteria metabolize citrate in the presence of carbohydrate (Walstra et al. 2005). Table 2 shows that citrate is metabolized in kefirs produced by plant‐based lactic acid bacteria. Citrate concentration increased on the first day of storage with the addition of almond skin. The citric acid content in the control and A coded sample increased significantly on the 7th day of storage (*p* < 0.05) and decreased on the 14th day. In samples coded B and C, there was no significant change during storage. These results showed that the increase in almond skin ratio has an inhibitory effect on the formation and fermentation of citric acid. It is thought that the change in organic acid content values is the result of metabolic activities of microorganisms due to the increase in the amount of added almond skin. As kefir has a complex flora, the organic acid produced by one group of microorganisms can be used as a substrate by another group of microorganisms in kefir (Bulat and Topcu 2021). In a different study on almond kefir, lactic acid was reported as 1.99 g/L and acetic acid as 0.61 g/L (Ustaoğlu‐Gençgönül et al. 2024), while lactic acid and acetic acid concentrations in different plant‐based water kefirs were reported as 0.02–4.81 g/L and 0.02–1.90 g/L, respectively. The organic acids have an important effect on many properties of fermented beverages, especially the characteristic taste and aroma (Tavares et al. 2023). Our findings in this study were consistent with these values (Table 2).

### 3.3 Total Phenolic Content (TPC) and Antioxidant Properties (DPPH)

**FIGURE 2:**
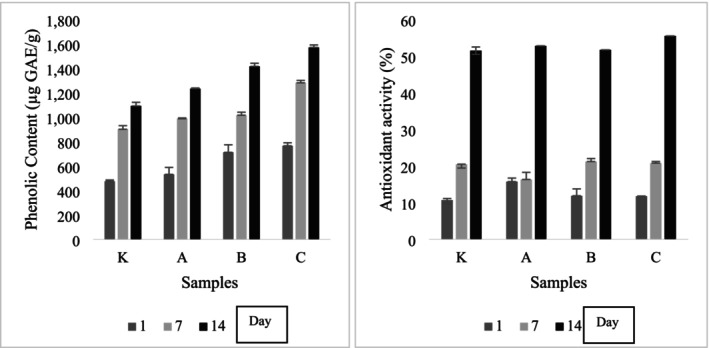
Total phenolic compound amounts (μg GAE/g) and antioxidant activity (%) of almond kefir samples during storage. K (0.15% plant‐based kefir starter culture + 1% Sugar + 0.1% salt + 4% maltodextirin + 1% gumarabic). A (0.15% plant‐based kefir starter culture + 1% Sugar + 0.1% salt + 4% maltodextirin+1% gumarabic+1% almond skin). B (0.15% plant‐based kefir starter culture + 1% Sugar+0.1% salt+4% maltodextirin+1% gumarabic+3% almond skin). C (0% 15 plant‐based kefir starter culture + 1% Sugar + 0.1% salt + 4% maltodextirin + 1% gumarabic + 5% almond skin) Days.

The almond skin used in almond kefir production contained 250.54 μg GAE/g of phenolic compounds. As shown in Figure 2, higher levels of total phenolic compounds were found in products with added almond skin, increasing with skin content. Phenolic compound levels increased in all samples during storage, with the highest average values observed in sample C, followed by B, A, and K. This trend aligns with previous studies that have reported variations in total phenolic compounds based on microorganisms and heat treatment methods (Wansutha et al. 2018). Although almond skin constitutes approximately 4%–8% of almonds, studies have reported that it contains 60%–80% of total phenolic compounds and phytochemicals such as phytosterol, phenolic acid, and polyphenolics in almonds are more concentrated in the skin of almonds (Chen et al. 2006).

In order to evaluate the antioxidative activity of almond kefir samples, the mean values with the DPPH method during the 14‐day storage period are given in Figure 2. The antioxidant activity of the samples, presented in Figure 2, displayed different peaks for each sample during storage. Sample A exhibited the highest activity on Day 1, sample B peaked on Day 7, and sample C demonstrated the highest activity on Day 14. Across all samples, antioxidant activity increased during storage, correlating with the rise in phenolic compounds. Almond extracts have been proven to have high metal ion chelation activity due to antioxidant polyphenols.

The results of phenolic compounds showed that the most important group of antioxidants and the antioxidant activity values of the samples to which almond skin was added are parallel. The fermentation process itself may contribute to the increased antioxidant activity. Some lactic acid bacteria produce enzymes, such as feruloyl esterases, that enhance the release of antioxidant phenols during fermentation with almond. This mechanism has been documented in previous studies and may explain the observed increase in phenolic content over time. Furthermore, research on fermented almond milk using various lactic acid bacteria (*S. thermophilus, L. delbrueckii* subsp. *bulgaricus*, *L. acidophilus*, and *B. animalis* subsp. *lactis*) has reported increased phenolic compound values over extended storage periods, attributed to bioactive compounds like β‐sitosterol and campesterol present in almond milk (Topçuoğlu and ve Ersan 2020). The significant contribution of phenolic compounds of almond skin to antioxidant capacity is well‐established, with studies indicating they possess triple antioxidant/antiradical activity (Prgomet et al. 2017). This property underscores the potential of almond skin as a valuable source of phenolic compounds and antioxidants in fermented almond milk products. The observed increase in benefits during storage and with higher skin content further supports the functional food potential of these products. These findings collectively highlight the synergistic effects of almond skin incorporation and fermentation on the phenolic content and antioxidant activity of almond kefir. Antioxidants are substances that prevent oxidation caused by free radicals in the body and capture and stabilize free radicals (Sarman and Gülle 2021). In a different study, it was shown that the addition of different bacteria ( *B. longum* and *B. lactis* ) to almond milk increased the phenolic and flavonoid content during storage compared to the control. Higher TPC and TFC are associated with higher free radical scavenging ability, which ultimately means that the antioxidant properties of fermented milk are improved (Sharma et al. 2021).

### 3.4 Microbiological Properties

The microbiological analysis of almond kefir samples over a 14‐day storage period revealed several interesting trends in bacterial and yeast populations (Table 3). Total Aerobic Mesophilic Bacteria (TAMB) counts ranged from 8.23 to 9.46 log cfu/mL^−1^, with the highest count in sample B and the lowest in sample C, though this difference was not statistically significant (*p* > 0.05).

**TABLE 3:** Microbiological properties of almond kefir samples (log cfu mL^−1^).

Sample	Storage (day)	TAMB	*Lactococcus* spp.	*Lactobacillus* spp.	*Lactobacillus acidophilus*	*Bifidobacterium bifidum*	Yeast
K	1	8.67 ± 0.07	8.07 ± 0.16	8.19 ± 0.01	4.15 ± 0.15	4.19 ± 0.35	2.74 ± 0.16
	7	8.44 ± 0.26	8.11 ± 0.00	8.69 ± 0.09	3.80 ± 0.10	4.75 ± 0.45	2.87 ± 0.87
	14	8.66 ± 0.06	8.42 ± 0.06	8.65 ± 0.00	3.98 ± 0.11	4.46 ± 0.34	2.48 ± 0.00
A	1	8.43 ± 0.02	7.59 ± 0.11	7.94 ± 0.24	4.24 ± 0.54	4.35 ± 0.35	3.50 ± 1.10
	7	8.24 ± 0.20	8.11 ± 0.03	8.55 ± 0.07	4.28 ± 0.20	4.72 ± 0.02	3.84 ± 0.06
	14	8.54 ± 0.16	8.08 ± 0.18	8.41 ± 0.29	4.30 ± 0.30	4.18 ± 0.07	3.52 ± 0.05
B	1	9.46 ± 1.08	7.39 ± 0.09	7.65 ± 0.65	4.64 ± 0.22	4.84 ± 0.06	4.26 ± 0.51
	7	8.23 ± 0.05	8.01 ± 0.17	8.21 ± 0.09	4.28 ± 0.02	5.41 ± 0.29	4.52 ± 0.04
	14	8.26 ± 0.18	8.48 ± 0.37	8.20 ± 0.08	4.52 ± 0.01	3.98 ± 0.20	3.78 ± 0.04
C	1	8.31 ± 0.20	8.17 ± 0.17	8.15 ± 0.15	4.64 ± 0.07	5.00 ± 0.26	4.76 ± 0.42
	7	8.30 ± 0.07	8.01 ± 0.11	8.24 ± 0.16	4.14 ± 0.54	4.85 ± 0.15	4.79 ± 0.01
	14	8.32 ± 0.00	8.14 ± 0.06	8.24 ± 0.04	4.37 ± 0.29	4.69 ± 0.32	3.15 ± 0.15

*Note:* K (0.15% plant‐based kefir starter culture +1% Sugar +0.1% salt +4% maltodextrin +1% gumarabic). A (0.15% plant‐based kefir starter culture +1% Sugar +0.1% salt +4% maltodextrin+1% gum arabic +1% almond skin). B (0.15% plant‐based kefir starter culture +1% Sugar +0.1% salt +4% maltodextrin+1% gumarabic +3% almond skin). C ((0%) 15 plant‐based kefir starters +1% Sugar +0.1% salt +4% maltodextrin +1% gumarabic +5% almond skin). The absence of lettering indicates that the difference between the samples is insignificant (*p* > 0.05).

*Lactococcus* spp. counts showed variable patterns across samples and storage times. Sample C had the highest count on Day 1, while samples K and A peaked on Day 7, and sample B on Day 14. Overall, the highest average lactococci count was observed on Day 14, with significant differences between storage days (*p* < 0.05). The stability of *Lactococcus* spp. numbers during storage is attributed to the nutritional composition of almond milk, including fiber, protein, and polyphenols, which support probiotic growth (Monagas et al. 2007). Yeast levels in almond milk kefirs were similar to those observed in water kefir made with plant‐based compounds and kefir made from cow's milk in previous studies (Ustaoğlu‐Gençgönül et al. 2024). Our results confirm that plant‐based kefir culture thrives in a plant‐based extract medium.

Regarding *Lactobacillus* spp., the control sample (K) consistently showed the highest counts, while sample B had the lowest average count throughout storage. This is thought to be due to the potential prebiotic effect of arabinose found in the cell wall pectin substances of almonds (Sethi et al. 2016). In addition, the xylooligosaccharide (XOS) in almond milk can be fermented by various strains of *Lactobacillus* and *Bifidobacterium* (Domínguez‐Murillo and Urías‐Silvas 2024).

This suggests that the addition of almond skin did not significantly impact lactobacilli numbers in almond kefirs. The differential growth rates between *Lactococcus* and *Lactobacillus* species may be related to their ability to metabolize sucrose, the primary sugar in almonds. Similarly, in a study conducted by Huang et al. (2023), the growth of different lactic acid bacteria in the fermentation of almond milk was followed, and it was reported that *Lactococci* caused rapid acidity reduction, *and Lb. bulgaricus* caused slower acidity reduction. The researchers reported that this was due to the fact that the sugar source contained in almonds is based on sucrose. It is thought that the presence of two uptake systems for sucrose in *Lactococcus* genus bacteria, phosphotransferase system and non‐PTS system (Reid and Abratt 2005) may lead to this difference.

*Lactobacillus acidophilus* counts were lowest in the control sample during storage. Samples B and C showed the highest *L. acidophilus* counts on Day 1, while samples A and B peaked on Day 7, and sample B maintained the highest count on Day 14. This increase in *L. acidophilus* numbers with the addition of almond skin suggests a potential prebiotic effect. Previous studies on almond skin and probiotics have also shown that it promotes the growth of *Bifidobacterium* spp. and *Lactobacillus* spp. (Al Zahrani and Shori 2023).

*Bifidobacterium bifidum* counts were highest in sample C on Days 1 and 14, while sample B showed the highest count on Day 7. These results support previous findings by Mandalari, Faulks, et al. (2010) indicating that almond skin may act as a prebiotic, enhancing probiotic bacterial growth. However, the current study revealed that this effect varies with the concentration of added almond skin and storage. Supporting these results, Barral‐Martinez et al. (2021) reported that almond skin contains bioactive prebiotic compounds such as xylooligosaccharides, polysaccharides, hemicellulose, and dietary fiber, which can enhance the growth of Bifidobacterium spp. during fermentation and storage.

Yeast counts in the almond kefir samples ranged from 2.48 to 4.79 log cfu mL^−1^. Samples containing almond skin consistently showed higher yeast counts compared to the control, with significant differences between samples (*p* < 0.05). This increase in both yeast and lactic acid bacteria content in samples with almond skin may be attributed to symbiotic interactions between yeasts and lactic acid bacteria, as reported by Bulat and Topcu (2021). The reason for this symbiotic effect in samples with almond skin added is thought to be the prebiotic effect of the skin. Yeasts are known to provide growth factors such as vitamins and amino acids for lactic acid bacteria, while the end products of lactic acid bacteria metabolism serve as energy sources for yeasts in kefir (Viljoen 2001).

### 3.5 Color Measurement

Color is one of the most important food attributes in a consumer's appreciation of foods. The lightness of a food sample is an important factor influencing the preference of a food product by the consumer. The type, concentration, and production method of food ingredients are the main factors affecting these parameters (Tobolková and Durec 2023). The color values of almond kefir samples during the 14‐day storage period are presented in Table 4. L* values decreased significantly with increasing almond skin ratios (*p* < 0.05). In other words, the control sample without skin exhibited the highest whiteness. Whiteness decreased in other samples as skin ratio increased, attributed to the brown color of almond skin. For each sample, L* values increased during storage time. The a* value, indicating greenness (−) or redness (+), decreased in all samples except K during storage. Samples C, B, A, and K showed progressively higher average a* values, with skin addition causing significant changes (*p* < 0.05). B and C samples displayed significant increases in b* values (blueness‐yellowness) during storage (*p* < 0.05). Total color difference increased in all samples during the storage period, with significant variations between samples (*p* < 0.05). Sample C showed the highest color difference value, followed by samples B and A. Almond skin amount likely contributed to these differences. The total color difference value (∆*E*), representing human‐discernible color distinctions, differed significantly among all samples (*p* < 0.05). Manzoor et al. (2021) reported that the L* value of raw almond milk was 64.38 and 67.75 in pasteurized almond milk, and the total color difference was 5.12 in pasteurized almond milk. In a different study, it was determined that the L* value of raw almond milk was 87.83 and the L* value of fermented almond milk was 90.48 on the first day of fermentation (Bernat et al. 2014). In a study on almond kefir, it was reported that the L* value was 76–77, the a* value was between 0 and 1, the b* value was between 6 to 7, and the total color difference (ΔE) was between 18 to 22 (Çomak Gocer and Koptagel 2023).

**TABLE 4:** Color values of almond kefir samples (mean ± SE; *n* = 2).

Sample	Storage (day)	L*	a*	b*	ΔE
K	1	78.20 ± 0.10^A^	0.00 ± 0.10^D^	7.35 ± 0.05^C^	
	7	78.65 ± 0.15^A^	0.15 ± 0.05^D^	7.40 ± 0.00^C^	
	14	79.00 ± 0.00^A^	0.15 ± 0.05^D^	7.25 ± 0.05^C^	
A	1	65.85 ± 2.25^B^	4.35 ± 0.55^C^	10.70 ± 1.20^B^	13.53 ± 2.66^C^
	7	68.05 ± 0.25^B^	3.95 ± 0.15^C^	9.90 ± 0.50^B^	11.54 ± 0.54^C^
	14	68.70 ± 0.40^B^	3.75 ± 0.05^C^	9.60 ± 0.40^B^	10.71 ± 0.15^C^
B	1	57.95 ± 2.65^C^	7.05 ± 0.85^B^	12.85 ± 0.65^A^	22.14 ± 2.99^B^
	7	60.75 ± 0.35^C^	6.15 ± 0.25^B^	12.30 ± 0.30^A^	19.51 ± 0.63^B^
	14	60.63 ± 0.57^C^	6.00 ± 0.10^B^	12.10 ± 0.40^A^	19.88 ± 0.66^B^
C	1	55.75 ± 0.55^D^	7.45 ± 0.15^A^	13.40 ± 0.10^A^	24.42 ± 0.71^A^
	7	55.80 ± 0.40^D^	7.55 ± 0.15^A^	13.60 ± 0.40^A^	24.81 ± 0.36^A^
	14	56.20 ± 0.40^D^	7.20 ± 0.10^A^	13.15 ± 0.15^A^	24.58 ± 0.43^A^

*Note:* K (0.15% plant‐based kefir starter culture +1% Sugar +0.1% salt +4% maltodextrin +1% gumarabic). A (0.15% plant‐based kefir starter culture +1% Sugar +0.1% salt +4% maltodextrin +1% gum arabic +1% almond skin). B (0.15% plant‐based kefir starter culture +1% Sugar +0.1% salt +4% maltodextrin+1% gumarabic +3% almond skin). C ((0%) 15 plant‐based kefir starters +1% Sugar +0.1% salt +4% maltodextrin +1% gumarabic +5% almond skin). Capital letters next to the values indicate that the difference between the samples is significant. (*p* < 0.05).

### 3.6 Dynamic Rheological Tests

Rheological results showed that the samples had pseudoplastic flow according to n values (Table 5). The Herschel‐Bulkley flow model was used in this study (*R* ^2^ = 0.9828–0.9998). The highest consistency index value among the samples was determined in sample K (Table 5). While samples K and C had the highest consistency index on the 7th day of storage, samples A and B had the highest consistency index on the 14th day. There was an interaction between the samples and storage, and it was observed that the control sample was thicker than the kefirs with added almond skin (*p* < 0.05). In a similar study on the rheological properties of a fermented product made from almond milk, it was stated that almond protein concentration, particle size, network structure, and interaction of fat components should affect functional properties and help optimization (Devnani et al. 2022). Almond milk protein concentration is a critical factor for gel structuring in the product (Devnani et al. 2020). In addition, in the development of rheological properties of kefir samples in our study, the fiber structure of vegetable milk and almond bark extract may affect the stability of the physical structure of the product (Devnani et al. 2022). In this study, since the fat content of the control kefir sample was higher than the samples with almond skin, it is thought that the consistency index value was higher in the control samples.

**TABLE 5:** Dynamic rheological values during storage of almond kefir samples (mean ± SE; *n* = 2).

Rheological properties	Storage time (days)	K	A	B	C
Consistency index K (Pa.s)	1	2.02 ± 0.20^B^	0.64 ± 0.04^DE^	0.57 ± 0.03^E^	1.01 ± 0.01^CDE^
	7	3.34 ± 0.04^A^	0.71 ± 0.04^DE^	0.84 ± 0.06^CDE^	1.24 ± 0.04^CD^
	14	3.21 ± 0.26^A^	1.43 ± 0.08^bc^	0.86 ± 0.00^CDE^	0.60 ± 0.01^E^
Flow behavior index (*n*)	1	0.37 ± 0.02	0.45 ± 0.01	0.45 ± 0.01	0.41 ± 0.01
	7	0.34 ± 0.05	0.46 ± 0.01	0.41 ± 0.01	0.35 ± 0.03
	14	0.36 ± 0.01	0.40 ± 0.01	0.37 ± 0.00	0.42 ± 0.01
Yield stress	1	0.35 ± 0.31	0.71 ± 0.21	0.50 ± 0.17	0.08 ± 0.36
	7	0.66 ± 0.67	0.56 ± 0.02	0.37 ± 0.03	0.09 ± 0.12
	14	0.65 ± 0.15	0.03 ± 0.56	0.04 ± 0.00	0.03 ± 0.00

*Note:* K (0.15% plant‐based kefir starter culture +1% Sugar +0.1% salt +4% maltodextrin +1% gumarabic). A (0.15% plant‐based kefir starter culture +1% Sugar +0.1% salt +4% maltodextrin +1% gum arabic +1% almond skin). B (0.15% plant‐based kefir starter culture +1% Sugar +0.1% salt +4% maltodextrin +1% gumarabic +3% almond skin). C ((0%) 15 plant‐based kefir starters +1% Sugar +0.1% salt +4% maltodextrin +1% gumarabic +5% almond skin). Interactions were detected between samples and storage days, and the differences between the values were shown with different capital letters (*p* < 0.05). The absence of lettering indicates that the difference between the samples is insignificant (*p* > 0.05).

As seen in Table 5, the samples with almond skin showed a higher flow behavior index than the control sample. It was determined that there was no significant difference between the samples and storage days of kefirs obtained from almond milk in terms of flow index (*p* > 0.05). In a study in which sucrose and maltose were added to a yogurt‐like fermented product from almond milk, it was found that viscosity increased in products with added carbohydrates during storage and decreased in products without added carbohydrates (Kavas and Kavas 2022). Starter cultures used in the production of fermented milk products play multifunctional roles during fermentation. It is reported that the low level of carbohydrate content in the composition of almond milk affects the development of acidity, prolongs the gelling time, and a non‐viscous product is obtained (Chang and Stone 1990). Therefore, it is a necessity to add carbohydrates in the production of fermented products produced by almond milk by means of starter cultures (Bernat et al. 2014). However, it is thought that the pH value decreased in all kefir samples due to the progression of storage, the gel structure was weaker, and the use of plant‐based starter caused the flow index value to decrease.

Yield stress is the minimum stress required for flowing. Kefir samples have low yield stress because they have a less viscosity. Regarding the structural stability of the samples, it is seen that the control sample exhibited a more stability during storage (Table 5). Statistically, there was no significant difference between the samples and storage days (*p* > 0.05). The sample coded C, which had the highest skin content, was the sample with the weakest stability and had the lowest yield stress during storage. Yield stress values of the samples coded A and B were the most dramatically decreased during storage.

### 3.7 Sensory Scores of Almond Kefir Samples

**FIGURE 3:**
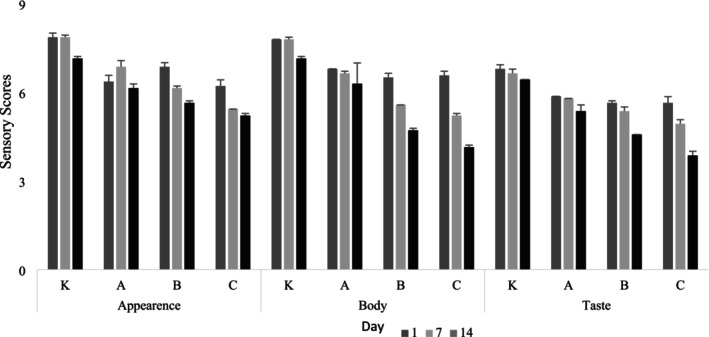
Sensory evaluation scores of almond kefir samples during storage. K (0.15% plant‐based kefir starter culture + 1% Sugar + 0.1% salt + 4% maltodextirin + 1% gumarabic). A (0.15% plant‐based kefir starter culture + 1% Sugar + 0.1% salt + 4% maltodextirin+1% gumarabic+1% almond skin). B (0.15% plant‐based kefir starter culture + 1% Sugar+0.1% salt+4% maltodextirin+1% gumarabic+3% almond skin). C ((0%) 15 plant‐based kefir starter culture + 1% Sugar + 0.1% salt + 4% maltodextirin + 1% gumarabic + 5% almond skin).

Sensory evaluation results, using human senses as instruments to measure quality and hedonic responses to stimuli (Meilgaard et al. 2016), are presented in Figure 3. Panelists' appearance scores for kefir samples showed significant differences between samples and storage days (*p* < 0.05). Sample K received the highest appearance score on all storage days. In terms of appearance, the samples with the highest shell content, coded C, received the least ratings. It was revealed that the kefir appearance was not liked by the panelists due to the increase in the shell ratio added. Similarly, the sample with code K received the highest value in structure and taste scores. Body scores decreased significantly during storage within samples (*p* < 0.05). Sample C, containing the highest almond skin content, received the lowest scores in all parameters. Although the skin imparted a distinct flavor to the fermented product, negatively affecting sensory properties, it was found to be acceptable. When evaluated from this perspective, it was determined that samples A and B had sensory scores closer to the K‐coded samples. In the sensory evaluation of the yogurt study made from almond milk, it received moderate acceptance values similar to our study. The rationale for this was explained by considering the synthesis of volatile acetic acid that occurs after fermentation. This acid seems to transfer detectable vinegary, pungent, and acidic odors to fermented products (Bernat et al. 2014). Many current studies focus on sensory properties in plant‐based innovative products (Akalın et al. 2024; Hew et al. 2023). The sensory data obtained in this study suggest that almond kefir with functional properties can be improved in terms of flavor, appearance, and structure.

## 4 Conclusion

This study demonstrated that health‐beneficial probiotic bacteria maintained viability in vegan kefir microflora produced with plant‐based almond milk, skin, and kefir culture during 14 days of storage. Almond skin, which is rich in fiber and phenolic compounds, significantly influenced total phenolic content and antioxidant capacity of almond kefir samples. The sample containing the highest skin content (Sample C) exhibited the highest total phenolic compound and antioxidant capacity values. Phenolic compound values increased both within and between samples during storage. Almond skin also enhanced the viability of probiotic microorganisms, suggesting potential functional applications for almond waste. Mineral analysis revealed the highest potassium content in the control sample. However, the calcium amounts of the almond skin‐added samples were found to be higher than the control sample, and the highest calcium (Ca) content was found in Sample C, containing 5% skin. These findings indicate that almond kefir with almond skin and plant‐based kefir culture could serve as an innovative functional product for vegan consumers. These innovative nondairy kefirs with functional properties can be consumed by target groups such as vegetarians, lactose‐intolerant individuals, and those allergic to cow's milk protein. Considering the risk of protein intake deficiency and total antioxidant activity insufficiency of plant milks, almond kefir would be a good choice for these individuals. However, some modifications in mouthfeel and/or flavor could be made to improve its sensory acceptability and ensure a wide consumer acceptance. It is thought that this study will contribute to the literature in this sense by providing basic information.

## Author Contributions

**Ayşe Şahin:** data curation (equal), formal analysis (equal). **İlkay Buran:** conceptualization (equal), data curation (equal), formal analysis (equal), funding acquisition (equal), investigation (equal), methodology (equal), project administration (equal), supervision (equal), visualization (equal), writing – review and editing (equal). **Ceren Akal:** formal analysis (equal), investigation (equal), methodology (equal), visualization (equal), writing – review and editing (equal).

## Conflicts of Interest

The authors declare no conflicts of interest.

## Data Availability

The data that support the findings of this study are available from the corresponding author upon reasonable request.
